# Communicating Awareness About COVID-19 Through Songs: An Example From Ghana

**DOI:** 10.3389/fpubh.2020.607830

**Published:** 2021-01-18

**Authors:** Rachel G. A. Thompson, Jerry John Nutor, Julene K. Johnson

**Affiliations:** ^1^Language Center, College of Humanities, University of Ghana, Accra, Ghana; ^2^Family Health Care Nursing Department, School of Nursing, University of California, San Francisco, CA, United States; ^3^Institute for Health and Aging, School of Nursing, University of California, San Francisco, CA, United States

**Keywords:** infectious disease, edutainment, preventive measure, song lyrics, multilingual population

## Abstract

Research has shown that music can be used to educate or disseminate information about public health crises. Grounded in the edutainment approach, we explored how songs are being used to create awareness about COVID-19 in Ghana, a sub-Saharan African country. YouTube was searched, and 28 songs met the study inclusion criteria. We conducted a thematic analysis of the song lyrics. Most lyrics were in English, Ghanaian Pidgin English, Akan, Ga, or Dagbani. Reflecting the multilingual population of Ghana, half of the songs contained three languages to convey their message, and only five songs were in one language. Eight themes emerged from the analysis: public health guidelines, COVID-19 is real and not a hoax, COVID-19 is infectious, prayer as method to stop the virus, emotional reaction and disruption of “everyday” activities; verbally expelling the virus, call for unity and collective efforts, and inspiring hope. We show that songs have the potential as a method for rapidly sharing information about emerging public health crises. Even though, it is beyond the scope of this study to draw conclusions about the reception and impact of songs on awareness and knowledge, the study shows that examining song lyrics can still be useful in understanding local attitudes toward COVID-19, as well as strategies for promoting preventive behaviors. We note that additional multidimensional efforts are needed to increase awareness among the general public about the COVID-19 pandemic.

## Introduction

On January 30, 2020, the World Health Organization (WHO) announced a “Public Health Emergency of International Concern” related to the spread of a novel coronavirus (SARS-CoV-2) that causes COVID-19. Shortly thereafter on March 12, 2020, the WHO declared COVID-19 a pandemic (1). Following these declarations and other reports about the emergence of a new severe respiratory infection, public health officials rapidly developed and deployed public health messages about the pandemic around the world. Over a short period of time, the general public was provided information about COVID-19 and guidance about how to mitigate spread of the virus (e.g., face coverings, washing hands, physical distancing). However, awareness (attitudes, knowledge) of COVID-19 differs across various parts of the world. For example, low awareness and misperceptions about COVID-19 were reported in some parts of the world, such as North America, Europe and Africa (2, 3). As a result, there have been urgent calls to find innovative ways to increase awareness about COVID-19 and help reduce the outbreak of this infectious disease (4, 5).

Ghana recorded its first two cases of COVID-19 on March 12, 2020 and cases have risen exponentially in the country to 13,717 as of June 20, 2020 (with 85 deaths), prompting presidential directives to increase awareness among citizens by creating and disseminating information on various preventive measures. These directives, mainly in English with direct translation in Ghanaian Sign Language, were disseminated on electronic and social media platforms. However, citizens who do not understand the national or official language must rely on other citizens to be accurately informed. As of April 25, 2020, the National Commission for Civic Education (NCCE), which could effectively educate citizens in every district using various indigenous Ghanaian languages, had been unable to do so due to lack of government funds (6). To compensate for the lack of funding, citizens were educated with regularly posted updates on the NCCE website and social media platforms (7). This strategy, however, does not address citizens who are more isolated and lack access to the internet or citizens who cannot read the official language.

Apart from the updates from the NCCE, some musicians in the country took personal initiatives to compose songs in local languages to educate the public about the disease. These songs were without any government or non-governmental organization support and were also not a part of an official campaign or intervention. Music plays an integral role in the culture of many African countries including Ghana. In addition to serving entertainment purposes, music highlights socio-cultural values and helps in the performances of daily routines. It is also used to recount history, and it forms a part of festivals, ceremonies related to rites of passage and other cultural functions (55). Music can be used as a vehicle for praise and criticism, especially in cases where authority figures are involved (8, 9). Also, it has the ability to alter knowledge, attitudes and behavior (10–13), and the potential to provide a way to access target audiences that could be missed with other methods of communication (14). Broadly speaking, music can play a pivotal role in helping increase awareness or gaining insight into different social issues.

Music forms a part of edutainment, a popular strategy that allows for educational messages to be embedded in entertainment media in order to positively change behaviors and attitudes (15). Edutainment is a combination of education and entertainment (57). It is a theory-based communication process which, according to Ganeshasundaram and Henley (52), “involves the design and implementation of media programs that deliberately incorporate persuasive, educational content in popular entertainment formats to influence audience knowledge, attitudes, behavioral intentions, and practices” (p. 311). This communication process is a tool for implicit persuasion as it is able to suspend disbelief and facilitate changes in the behavior of an audience (16, 17). Govender (53) asserts that in terms of facilitating a desired behavioral or social change, edutainment has been more innovative in Africa than in other parts of the world. As a result, some non-governmental agencies financially support programs and activities that engage members of a community and attempt to solve social problems through creative artistic processes that are receptive and entertaining (18).

Generally, edutainment health interventions are complementary to traditional public health interventions (18). Several studies have reported how songs and music can be used as a means of education and increasing awareness in public health context and clinical experience (12, 19–21, 50). Thus, the use of music to influence knowledge, attitudes and behaviors toward infectious diseases like COVID-19 is not a new phenomenon in Africa. In relation to HIV/AIDS and Ebola, for instance, research shows how popular artists in the region incorporated health-promoting messages and basic information about these diseases, and communicated preventative measures in their songs (14, 22–24). In 2000, a campaign dubbed “Stop AIDS: Love Life” involved a music video produced by Ghanaian musicians to increase awareness about HIV/AIDS in the country. The campaign was implemented by Johns Hopkins University and the Ghana Social Marketing Foundation. It was aimed at de-stigmatizing HIV/AIDS, encouraging compassionate care and support for people living with HIV/AIDS, and encouraging citizens to adopt safe sex behavior (25). Also, various studies document how popular musicians were commissioned by organizations such as WHO and UNICEF in 2014–2016 to compose prevention songs as a response to the Ebola outbreak in Liberia and other parts of Africa (21, 26).

Certain characteristics of songs, such as repetition and the capacity for involuntary recall, enhance the effectiveness of educational messages (27). These characteristics underpin reasons why, as earlier mentioned, HIV/AIDS and Ebola prevention messages were conveyed through songs in various parts of Africa (28–30). In Uganda, for example, music was recognized by healthcare workers as “a more localized and thus more effective medical intervention than outreach efforts in the form of lectures and seminars” ((29), p. 27). The assumption is that in African communities, the performance of songs can facilitate information dissemination and public debate on sensitive health topics (15). It is therefore not surprising that songs have been composed in Ghana and other African nations (e.g., Liberia, Nigeria, South Africa, Uganda, Cameroun, and Kenya) to educate people about the novel coronavirus (31). The purpose of this paper is to describe the content of song lyrics related to COVID-19 messaging and to highlight among other things the preventative measures embedded in them. The songs examined were composed by individual Ghanaian musicians and posted on their YouTube channels during the COVID-19 pandemic.

## Methods

### Setting

Ghana is located in West Africa, bordered on the west side by the Ivory Coast, and by the Atlantic Ocean in the south. According to recent census data, the population of Ghana is 29 million (32) with over 81 spoken languages (51). Four languages, including English, Akan, Hausa, and Ghanaian Pidgin English (GhPE), are the most common languages used for broad communication in the country (51).

Akan is the primary language spoken in the south (including the capital, Accra) and Hausa is the primary language spoken in the north (33). However, English is Ghana's official national language used in education, politics, government business, international trade, and technology (34–36). English is also the language used in print media and most electronic media programs. However, about eight of the written indigenous languages, including Akan, Ewe, Ga, Dagbani, Dagaare, and Gonja, are used for certain programs on radio and television (37, 38).

### Data Extraction

YouTube [www.youtube.com], a music sharing website, was searched to identify coronavirus-related songs from Ghanaian musicians. following Google. According to the 2020 Alexa report, following Google [www.google.com], YouTube is the second of 500 top websites frequently used among Ghanaians (www.alexa.com/topsites/countries/GH). This makes YouTube the most popular and most patronized music and video sharing website in Ghana. The search was conducted from April 1, 2020 through May 30, 2020 with the following terms “Ghana coronavirus song,” “coronavirus song Ghana,” “coronavirus song from Ghana,” “coronavirus audio from Ghana,” and “coronavirus video from Ghana.” A total of 40 songs from both well-established and upcoming musicians were found. We included songs by Ghanaian artists in English or Ghanaian local language with any COVID-19 educative information. We excluded songs that only mentioned COVID-19 without providing any implicit or explicit public health message about the disease. An example can be retrieved from https://www.youtube.com/watch?v=EduNrGL5OwE.

The songs were searched and selected by three bachelor-prepared research assistants based on the exclusion and inclusion criteria. The selected songs were then reviewed by two PhD prepared researchers. Each song was assessed solely in terms of its lyrics and no other features such as tempo, rhythm, or mood. Non-English lyrics were translated and transcribed by language experts from Ghana. The lyrics were then reviewed to obtain information about key issues raised (themes), language(s) used, and references to specific symptoms and preventive measures. Of the 40 songs identified, 28 met the inclusion criteria.

### Data Analysis

We conducted a thematic analysis to examine the lyrics of the selected songs. As a qualitative method, a thematic analysis allows for identifying, analyzing, and reporting patterned themes within the songs. The four phases of analysis (immersion, code generation, theme identification, and theme confirmation) that guide a systematic thematic analysis were employed (39). First, we became immersed and acquainted with the data by reviewing the lyrics of each song and noting preliminary points of interest. Secondly, two of the authors engaged with the transcribed lyrics and lyrics were double-coded. Codes were then compared to ensure consistency. We coded any mention of coronavirus, its effects, or how people can avoid it or deal with it. Thirdly, we merged codes and categorized them into major themes. Lastly, we reviewed and made clear definitions for each theme generated from the data. The most representative lyrics for each identified theme were selected to summarize the findings. Non-English lyrics are first presented in italics in the original language, and then followed by an English translation. There was no disagreement between the authors during the analysis.

## Results

The final dataset included 28 songs that met study selection criteria. In addition to the themes described below, other characteristics of the lyrics are summarized in Table 1, including the languages in which the songs were sung, the signs and symptoms mentioned in the lyrics, and any mention of coronavirus preventive measures. The songs were in various genres of Ghanaian music including Hip-life, Gospel and Highlife. While only five songs were in one language, most were multilingual, with 14 songs in three languages and 10 songs in two languages (See Table 1). The languages that dominated the songs were Akan (*n* = 21), English (*n* = 16), and Ghanaian Pidgin English (GhPE) (*n* = 13).

**Table 1:** Characteristics of the 28 songs.

**Song #**	**Language(s)**	**Themes**	**Symptoms**	**Safety and preventive measures**
1	English Akan Ewe	It is infectious; prayer; public health guidelines	–	Maintain social distancing; stop touching your eyes, nose, mouth; Clean surfaces frequently; wash your hands with soap under running water; sanitize your hand; cover your mouth when sneezing or coughing; wear a face mask; stop shaking hands; stop hugging
2	English GhPE Akan	Inspiring hope; public health guidelines; emotional reaction and disruption of everyday activities; prayer	–	Wash your hands; sanitize; stay home; use your face mask
3	GhPE Akan Hausa	It is real and not a hoax; it is infectious; public health guidelines	_	Hand sanitize; stay home; exercise; social distancing
4	GhPE Akan	Public health guidelines	_	Use sanitizer; wash your hands with soap; drink water regularly; use face mask; Social distancing; stay home
5	Akan English	Public health guidelines	–	No handshaking; social distancing; wash your hands; use sanitizer; go to the hospital if you are not feeling well; don't touch your nose, face, eyes; Stay home
6	GhPE Akan English	It is infectious; Inspiring hope; call for unity and collective efforts; public health guidelines	–	Wash your hands; sanitize your hands; try not to touch your face; social distancing; stay indoors
7	Sisala Waale English	Public health guidelines	–	Cough into your elbow or a handkerchief; sneeze into a handkerchief; wash your hands with soap; Stay home; don't go to funerals or to the beach
8	English Akan	Public health guidelines; call for unity and collective efforts	–	Use sanitizer; wash your hands under running water with soap; avoid handshakes; sneeze and cough into handkerchief; avoid social gatherings; practice social distancing; Stay home
9	Akan Ga English	Public health guidelines; verbally expelling the virus	–	Wash your hands well: use a mask to cover your nose; Stay indoors; let's be clean
10	English Akan	It is real and not a hoax; prayer; public health guidelines	–	Stay indoors; wash your hands with soap and water; use hand sanitizer; avoid touch people
11	Akan English	It is real and not a hoax; prayer; public health guidelines	–	Wash your hands with soap; use sanitizer; remember to wear face mask when going out; practice social distancing
12	GhPE	It is infectious; inspiring hope; public health guidelines	–	User hand sanitizer; wash your hands with soap; wear mask; stay in your room
13	Akan	It is infectious; public health guidelines; prayer; call for unity and collective efforts	–	No handshake; social distancing; use tissue to cover your mouth when coughing; wash your hand under running water; use sanitizer
14	GhPE Akan	It is real and not a hoax; verbally expelling the virus; public health guidelines; prayer	–	Go everywhere with your hand sanitizer; Avoid touch your eyes, mouth, nose; wash your hands with soap and water; stop touching undisinfected surfaces
15	English GhPE	It is real and not a hoax; public health guidelines	Coughing; Sneezing; Sore throat	No handshake; use soap and running water to wash your hands; use alcohol-based sanitizer; keep yourself clean; maintain social distance; if you are coughing, sneezing or have sore throat, stay home and call your doctor
16	GhPE Dagbani	It is real and not a hoax; prayer; it is infectious; public health guidelines	High temperature; Coughing	Use sanitizer; no handshake; isolate people who are sick
17	Akan English	It is real and not a hoax; emotional reaction and disruption of everyday activities; prayer; public health guidelines		Wash your hands with soap; use hand sanitizer; no handshaking; move away from anyone sneezing or coughing; cover your nose and mouth
18	Akan English	It is real and not a hoax; it is infectious; public health guidelines; prayer	High temperature (38–40°C); Running nose; Persistent cough; Difficulty in breathing; Sneezing; Body weakness	Boost your immune system by eating food that contains vitamin C and A, and zinc; keep your surroundings clean; wash your hand with soap; avoid handshakes; sneeze into a tissue or handkerchief; cover your mouth while coughing; cough into your elbow when you have no handkerchief or tissue; dispose of used tissue; Practice social distancing; avoid touching your mouth, ears, eyes, and nose; avoid touching surfaces; use face mask; stay home
19	Akan Ga	It is real and not a hoax; it is infectious; public health guidelines; prayer	Sneezing; Difficulty in breathing; Coughing; Running nose	No handshaking; keep 2-meters away when chatting; don't touch your face; wash your hands; use alcohol-based sanitizer; when coughing or sneezing, cover your mouth; when you have running nose, coughing; sneezing, difficulty in breathing, go to the hospital for a test or call a health worker; stay at home when you are not feeling well; when you return from a trip abroad, allow to be quarantined
20	GhPE	It is real and not a hoax; public health guidelines	Coughing; Headache	Stay indoors; wash your hands with soap; cover your nose; use alcoholic-based hand sanitizer; keep 2-meters away from anyone; don't touch your face, nose, eye
21	English Akan	It is real and not a hoax; it is infectious; public health guidelines; prayer	–	Stay home; wash your hands with soap and water for a min of 20 secs; social distancing
22	English Akan Ga	It is real and not a hoax; It is infectious; emotional reaction and disruption of everyday activities; verbally expelling the virus; public health guidelines; inspiring hope	–	Don't touch your eyes, mouth, nose; wash your hands; wear face mask; Avoid social gathering (naming ceremony, funeral); stay home
23	English Akan Ga Hausa	It is real and not a hoax; public health guidelines; call for unity and collective efforts; inspiring hope; prayer	–	Be neat; wash your hands with soap and water; no handshake; no hugging; no outing; more bathing; quarantine
24	Akan GhPE Ga	It is real and not a hoax; prayer; public health guidelines	–	Wash your hand with soap under running water; use sanitizer; no handshaking; Stay home; don't touch your nose, eyes, mouth
25	GhPE Akan	It is real and not a hoax; prayer; public health guidelines	–	Wash your hand with soap under running water; clean your hands with hand sanitizer; no handshaking; avoid party; no church going, no mosque going; when coughing or sneezing, cover your mouth
26	Akan	It is infectious; emotional reaction and disruption of everyday activities; public health guidelines; prayer	–	Wash your hand with soap under running water; use sanitizer; avoid social gatherings
27	GhPE Ga	It is real and not a hoax; verbally expelling the virus; emotional reaction and disruption of everyday activities; public health guidelines	–	Wash your hand with soap under running water; use sanitizer
28	English GhPE	It is real and not a hoax; it is infectious; emotional reaction and disruption of everyday activities; public health guidelines; call for unity and collective efforts	–	Wash your hand with soap and water; avoid handshakes; don't touch your face; Remember your sanitizer; cover your mouth while coughing; avoid touching your face; stay home

As shown in Table 1, all 28 songs mention some way to help prevent the infection. The step that people can take to prevent the transmission of the disease were also integrated in the lyrics. Examples of prevention included: wash your hands with soap under running water; rub your hands with alcohol-based sanitizer on a regular basis; avoid handshakes; use face masks; avoid social gatherings such as parties, funerals, and outdoor ceremonies; cover your mouth with a tissue when coughing or sneezing; and put yourself at a distance of about 2 m (6 feet) from other people during interactions.

Eight major themes emerged from the analysis of the song lyrics: (1) Public health guidelines, (2) COVID-19 is real and not a hoax, (3) COVID-19 is infectious, (4) Prayer as method to stop the virus, (5) Emotional responses and disruption of “everyday” activities, (6) Verbally expelling the virus, (7) Call for unity and collective efforts, and (8) Inspiring hope. Each of these themes is described below.

### Public Health Guidelines

The most prevalent theme in all the songs was emphasis on public health guidelines including the need for personal responsibility to avoid getting infected and infecting others. The lyrics suggested that the only way to combat the disease is to adhere to public health guidelines. Lyrics indicated that people can generally take care of themselves during the pandemic by adopting healthy lifestyles and by being compliant with public health guidelines from the WHO, Ghana Health Service, and Ministry of Health. Examples of lyrics that support this theme are as follow.

Lyrics supporting the theme of public health guidelines

**Table:** 

**Original Lyrics**	**English Translation of the Lyrics**
*If you wanna stay alive stay home I am here to advice ya My dear please use sanitizer* *Wash your hands under running water with soap and be wise Avoide handshake Sneeze and cough into handkie. Social gathering onua menk menk nfa nsem pi mma. Stay at home and be safe na practice social distancing eh. Let's help the government fight the corona* **(#8) GhPE and Akan**	“If you want to stay alive stay home I am here to advice you. My dear, please use sanitizer Wash your hands under running water with soap and be wise Avoid handshakes. Sneeze and cough into a handkerchief. Social gathering is not necessary, don't go and bring trouble. Stay at home and be safe and practice social distancing eh. Let's help the government to fight the coronavirus”
Coronavirus is dangerous. Covid-19 is deadly. Coronavirus is a common enemy to the world so beware. Let's maintain our social distancing. Stop touching your eyes, your nose, stop touching your mouth. Beware beware my sister. Beware my brethren beware. It's dangerous, beware **(#1) English**	
*Baby take care of yourself before it's too late I told ya Coromental Coro danger* *Coro no good Coro too bad Coro Coro Coro Coro too much Coro deadly* *Coro virus Coro mess up Coro Coro Coro Coro* **(#3) English and GhPE**	“Baby take care of yourself before it's too late. I told you. Coronavirus is dangerous. Coronavirus is not good. Coronavirus is too bad. Coronavirus is prevalent. Coronavirus is deadly Coronavirus has messed up everything”
*Regularly drink water. If you're coughing or sneezing nose mask bi the new anthem* *nnye saa wobe da ntem* *Social distancing too dey hia. Chale stay home is the best option me nuaa* *Exercise the body and eat healthy* **(#4) English, GhPE and Akan**	“Regularly drink water. If you're coughing or sneezing, nose mask is the new anthem or else you will die. Social distancing is very important. My friend, staying home is the best option. Exercise the body and eat healthy”
*Yen b yen ho ban. Yen hwε yen ho yiye. Yen di yen ho nni … Cover wa no sε wo cough*. *Kata wo hwene sε wo hwenten a. Nhyeamu ahorow no nso mo mma yengyae* **(#18) Akan**	“Let's protect ourselves. Let's take care of ourselves. Let's keep ourselves clean … Cover your mouth when you cough. Cover your nose when you sneeze. Let's avoid social gatherings”
Please stay home because of coronavirus. Don't go to funerals or to the beachside Hey, hey coronavirus, coronavirus. Coronavirus will be the death of you **(#7) English**	“Yeah if we live anyhow, then we may suffer, yeah, that is why I'm urging everyone. Friend, stay safe”
Alcoholic sanitizer for your hands Keep two meters away from anyone (#20) GhPE	“Keep alcoholic sanitizer handy. Keep two meters away from anyone”

As seen in Table 2, all the preventive measures were found in the song lyrics. Handwashing was the most frequently mentioned measure (*n* = 25). The musicians also emphasized the need for individual responsibility to avoid contracting coronavirus and subsequently, ward off death. They added that in order to maintain health, one must eat healthy, maintain good personal hygiene, keep surroundings clean, remain hydrated, and exercise regularly.

**Table 2:** Preventive measures as heard in song lyrics in order of frequency.

**Information**	**Frequency (%)**
Wash your hands	25 (18)
Use hand sanitizer	22 (16)
Stay home	19 (14)
Social distancing/Avoid social gathering	19 (14)
No handshake/hugging	15 (11)
Cover your mouth when sneezing/coughing	13 (9)
Avoid touching eyes, nose and mouth	10 (7)
Wear face mask	8 (6)
Isolate sick people	4 (3)
Clean/Disinfect surfaces regularly	3 (2)

### COVID-19 Is Real and Not a Hoax

The songs sought to inform the public that coronavirus is real and not a hoax as believed by some people. Some lyrics indicated that the disease emerged from China and spread to almost all parts of the world including Europe, United States of America, and Africa. Musicians sang about many people dying, and many others being hospitalized in these countries as a result of contracting the disease. Others referred to the fact that Ghana first recorded only two cases but there has since been an exponential growth in the number of infections. Lyrics that support this theme are listed below.

Lyrics supporting the theme that coronavirus is real and not a hoax

**Table:** 

**Original Lyrics**	**English Translation of the Lyrics**
*Yareε abae enye ntoro e no b joke. Enye agoro, hwε woho yie oo. Take precautions my people. Be vigilant* **(#14) Akan and English**	“This disease is not a hoax; it is not a joke. It is not a joke, take very good care of yourself. Take precautions, my people. Be vigilant”
*COVID-19 pandemic e shock the whole world like electric. Coronavirus is very dangerous so take am serious make you no joke at all Menfa di agoro. You never know you no go know you never know who dey carry go ooh Make you no joke at all* **(#25) GhPE and Akan**	“COVID-19 pandemic has shocked the whole world like electric. Coronavirus is very dangerous so take it seriously and don't joke at all. Don't play with it. You may never know; you will not know who has been infected. Don't joke at all”
*The spread is getting this serious. It started from Wuhan in China. Italy recording cases of the day. Spain is in pain … nnεra nkoaa na yε recorded cases two. Nanso bεhwε nnε more than* 132 **(#22) English and Akan**	“The spread is getting this serious. It started from Wuhan in China. Italy recording cases of the day. Spain is in pain … it was just yesterday we recorded two cases. But today, there are more than 132 cases”
*Alubu bunti paa tiηna? Ghana Ghana Ghana, Ghana Ghana Alubu bunti paa tiηna? Duru η, ti daa wumla diyala China kadi gungu ti paai tina Ghana. Ashebti shab dumi shab dumi N baye Pam kpemi, pam kpemi, pam kpemi N baye Dimalla nandahama* **(#16) Dagbani**	“What is this calamity that has befallen us? Ghana Ghana Ghana, Ghana Ghana What is this calamity that has befallen us? This is a disease we heard was in China and it has roamed and roamed, and it is now with us in Ghana. Some have been hospitalized, some have been hospitalized so many have died, so many have died, so many have died. It is sorrowful”
*Corona yi deε εde asem aba. Barima, ama yε sre a ehu ab yεn. Hwε, ehyε ase εw China. Na ananteε abe duru America. Yen deε anka nfa yεn ho o. Ɔman Ghana yε te yen bebi o. Nanso nnε aduru ha* **(#11) Akan**	“This coronavirus will bring issues. Man, it is making us scared. Look, it started from China, went to America. We, in Ghana, were minding our business but today it is here”
*Corona Corona, e kill more people for china; e go Italy, Verona, Burkina, over to Ghana.* *Corona Corona, e kill more people for America, from Belgium down to Nigeria, now down down into my area* **(#10) GhPE**	“Coronavirus Coronavirus, it has killed more people in china; It has gone to Italy, Verona, Burkina, over to Ghana. Coronavirus Coronavirus, it has killed more people in America, from Belgium down to Nigeria, now it is my area”

### COVID-19 Is Infectious

In addition to informing the public that COVID-19 is real and not a hoax, the songs warned that the virus is very infectious. Some lyrics made it clear that the novel coronavirus is even more infectious than other viral diseases including HIV, SARS, and Ebola, and older adults are the most vulnerable. The songs that projected this theme also had content about the disease affecting all categories of people, regardless of race, age, or social status. Below are examples of lyrics to support this theme.

Lyrics supporting the theme that coronavirus is infectious

**Table:** 

**Original Lyrics**	**English Translation of the Lyrics**
Wei nyε bacteria, wei εyε virus kyen bird flu, SARS εne Ebola **(#19) Akan**	“This is not bacteria, it is a virus, it is worse than bird flu, SARS, and Ebola”
Yarebae a w'abae nsuro bibini. Nsuro obroni, nsuro Indian ni. nsuro obia w wiase mu **(#21) Akan**	“This disease is not afraid of the blacks, not afraid of whites, not afraid of Indians, not afraid of anyone in this world (It can infect anyone)”
From China to Italy You dey kill around more than HIV. Africa mama eh Just tell your people to be careful Don't you panic just be careful. Just do needs and be careful **(#27) GhPE**	“From China to Italy, you are killing more than HIV. Africa mama eh, Just tell your people to be careful. Don't panic, just be careful. Just do the needful and be careful”
Corona virus yi deε εyε hu… ensuro obolo na ensure tsingilingi. Nsuro nnipa tenten na nsuro … Nfa ho sε wo ho yε fε anaa wo ho yε tan. The whole world one word, quarantine **(#22) Akan**	“Coronavirus, this is scary … It is not afraid of the fat person or afraid slim person It is not afraid of a tall person or afraid of … It doesn't matter whether you are beautiful or ugly The whole world, one word, quarantine”
Nidi degbaaga. Domini din gbaaga N baye din gbaagama. Dimi ni gbaama, din tuui lui so. Coronavirus zalizaa Corona beche bundan bii nandana. Debiche beble bii ninkurigu Din zuɤu, anyama tima nuu kambi dee. Dibi wuhi ni zilman kai kambi deeN gula nmang, ka gua zuɤu kambi dee **(#16) Dagbani**	“It should not infect you, because if it infects you, it will infect me. If it infects me, I can pass it to someone else. ‘Coronavirus has brought a difficult situation. Coronavirus does not leave out the rich or the poor; it does not leave out the child or the elderly so, if you see me and extend a shaking hand and I don't receive it, it does not mean it is out of disrespect. I have not received your handshake because I want to protect you and myself”

### Prayer as Method to Stop the Virus

More than half of the songs expressed the idea that divine intervention is needed to curb the spread of the virus, and to heal and protect people. Some of the lyrics urged their audience to pray to God. For example:

**Table:** 

**Original Lyrics**	**English Translation of the Lyrics**
*Yen sre Twedeampn na obehu yen mb* **(#17 Akan)**	“Let us plead with the Almighty and he will show us mercy”
*Yen su fre Nyame, obehu yen mb* **(#18 Akan)**	“Let us cry unto God, he will show us mercy”
*We for pray say Baba go come save* **(#23 GhPE)**	“We need to pray that the Father will save us”

Other lyrics were actual prayers asking God to help find a vaccine, restore calm to the world, or protect the people. The following are three such examples:

**Table:** 

**Original Lyrics**	**English Translation of the Lyrics**
*Deadly virus is spreading, oh my God in Heaven, abeg give us a vaccine to clear out this worldwide burden* **(#14 GhPE)**	“Deadly virus is spreading, oh my God in Heaven, please give us a vaccine to clear out this worldwide burden”
εpo ne asorkyeε wura, esum εyε duru yεn, kasa na εnyε dinn, kasa na εnyε komm, kasa na εnyε hann **(#26 Akan)**	“Owner of the sea and waves, darkness is covering us, speak and let it be silent, speak and let it be quiet, speak, and let there be light”
*Kpeηlan naawuni … Zaηmi a nam, yeko gulibu guliti* **(#16 Dagbani)**	“Almighty God … use your power, your protective ability to protect us”

### Emotional Reaction and Disruption of “Everyday” Activities

Apart from the health implications of coronavirus, other effects of the disease highlighted in the data include the fear it has induced in people and the emotional reactions to disruptions in social and economic activities. In the examples below, the world in general is identified as a fearful place, and some specific countries have been described as “ghost towns” due to the restrictions placed on movement.

**Table:** 

**Original Lyrics**	**English Translation of the Lyrics**
*Yareε yi yε hu o. Asia, Europe adane nsaman kurow No, no, no εnyε fadi agor o* **(#17) Akan**	“This disease is very scary. Asian and Europe have become like ghost towns. No, no, no, it is not a joke.”
Everybody, living in fear, hoping that this virus disappears **(#23) English**	
*Coro Aunty Coro, Mama mesuro.* *Coronavirus mesuro. Me deε mesuro* **(#24) Akan**	“Coro Aunty Coro, Mama, I am afraid Coronavirus, I am afraid, As for me, I'm afraid.”

Other lyrics mentioned that social gatherings are no longer allowed, thus no more public entertainment, sports, or religious activities. Consequently, people are feeling lonely because they cannot see their partners, loved ones or friends. Here are examples of lyrics that support this theme.

Lyrics supporting the theme of coronavirus emotional reactions to disrupted activities

**Table:** 

**Original Lyrics**	**English Translation of the Lyrics**
*You dey put my people for sorrow. We no fit come together no more. Social distancing is killing industry. We no fit come together and play showsWe no fit come together act movies. We no fit come together play footballWeytin dey happen? African dey panic. Europe dey panic. Americas dey panic, Asia dey panic* **(#27) GhPE**	“You are putting my people in sorrow. We are not able to come together anymore. Social distancing is killing our businesses. We are not able to come together for entertainment shows. We are not able to come together to act movies. We are not able to come together to play football. What is happening? Africa is panicking, Europe is panicking, America is panicking. Asia is panicking”
*I can't go out I can't go out I'm so alone nowI miss Bloombar I miss Bloombar I want to chill now. Want to bolingoooo I want to link up … Life start dey bore lately. What's on the news? Everyday, all day bad news soor* **(#6) English and GhPE**	“I can't go out, I can't go out, I'm so alone now I miss Bloombar, I miss Bloombar, I want to chill now. I want to have fun; I want to socialize … Life is boring lately. What's on the news? Everyday, all day, it is only bad news”
Hello COVID-19, w'ama m'afe me wifey. Home alone in loneliness …As fo aka dan mu; Asre bia aka dan mu **(#2) English and Akan**	“Hello COVID-19, you have made me miss my wife. I am home alone in loneliness … Pastors are stuck in their rooms; all the churches are no more open”

### Verbally Expelling the Virus

On the basis of belief in the power of words by Ghanaians in general, some musicians verbally expelled the virus in their lyrics. Some clearly stated the world does not want this disease, while others asked the virus to go back to where it came from. Four examples of this theme are presented below.

Lyrics supporting the theme of verbally expelling the virus

**Table:** 

**Original Lyrics**	**English Translation of the Lyrics**
*Coronavirus we taya, go back from you dey come from. You came in unannounced; you never tell nobody where you from* **(#27) GhPE**	“Coronavirus we are tired, go back to where you came from. You came in unannounced; you didn't tell anyone where you came from”
*Coronavirus yεmpε; sε Ghana ha, daabi Coronavirus yεnpε; sε Africa, daabi Coronavirus yεmpε; sε USA, yεmpε; Coronavirus daabi; sε Italy, yεmpε* ***(#9) Akan***	“Coronavirus, we don't want it in Ghana, no, Coronavirus we don't want it in Africa, no. Coronavirus we don't want it in USA, we don't want it; Coronavirus, no; in Italy, we don't want it”
*Coronavirus, hela nεε na waa ts … Hela nεε wsum wsum wsum kwraa* **(#9) Ga**	“Coronavirus, this disease is too strong … We don't want this disease, we don't want it, we don't want it at all”
*Oh yeah wherever you dey come from. I tell you say hey hey hey hey abeg make you lost–Yesu mogya ka w'anim. Abeg make you lost. Ogya ogya hyew w'anim. Coronavirus wherever you dey come from. I tell you say abeg make you lost* **(#14) GhPE and Akan**	“Oh yeah wherever you are coming from. I am saying, hey hey hey hey please get lost–May the blood of Jesus rebuke you. Please get lost. May fire burn you. Coronavirus, wherever you are coming from, I am telling you that, please, get lost”
Yεmpε, yεmpε, yεmpε, Corona yεmpεYεmpε o, yεmpε, yεmpε **(#22) Akan**	“We don't want it, we don't want it, we don't want it. We don't want Coronavirus. We don't want it, we don't want it, we don't want it”

### Call for Unity and Collective Efforts

In the face of the COVID-19 pandemic, many lyrics in the data have called for a more collaborative mindset among individuals at the national level and among nations of the world at the global level. As everyone struggles to adapt to the current reality, people must recognize the need to support and collaborate across borders, to share what works and what does not work in fighting the disease. Some musicians described coronavirus as “a common enemy” and thus reinforced the need for unity and collective efforts at both national and global level to help defeat the virus. Some examples of this theme are included below.

Lyrics supporting the theme that coronavirus requires unity and collective efforts

**Table:** 

**Original Lyrics**	**English Translation of the Lyrics**
Let's come together to stop Corona Corona. We can if we try and we can start all over **(#28) English**	
*Coro be enemy wanna enemy I call on the whole nation. Let's fight our enemy, our enemy,* *Coro be enemy. Ghana Ghana Ghana, Ghana Ghana* **(#16) GhPE**	“Coronavirus is an enemy, our enemy I call on the whole nation. Let's fight our enemy our enemy, Coronavirus is an enemy. Ghana Ghana Ghana, Ghana Ghana”
*Global war but everybro jie eye dey protect their own We should come together* *That's when we stand a chance so my friend This no bi time to point fingers. The thing dey spread, Corona* **(#6) GhPE**	“This is a global war, but everyone is focusing on protecting their own. We should come together That's when we stand a chance so my friend this is not the time to point fingers. The thing is spreading, Coronavirus”
It's time to love one another. It's time for the world to come together. No black no white no yellow. Cos nobody is promised tomorrow **(#23) English**	
*Presenters mo mb dawuro o, yenkyia biom TV stations ahorow nyinaa, yenkyia biom na yadeε ne ho yε hu yiye paa nti yenkyia biom oo o. Mo mb yadeε ne ho dawuro oo na yadeε ne ho yε hu yiye paa, yenkyia biom* **(#13) Akan**	“Presenters should spread the news, no more handshaking, all the different TV stations, no more handshaking. No more shaking of hands because the disease is very scary. Spread the news about the disease, for the disease is very scary, no more handshaking”

### Inspiring hope

There were also lyrics that assured their audience that this pandemic will not last forever, and that soon the world will recover from its negative impact. Some musicians also expressed their belief that God will heal those infected and deliver the world from this pandemic.

Lyrics that illustrate the theme of inspiring hope:

**Table:** 

**Original Lyrics**	**English Translation of the Lyrics**
*Nyame beyi yεn afiri mu, yareε ne amaneε wei mu* **(#22) Akan**	“God will deliver us from this sickness and trouble”
Sing Africa oh oh oh Sing America oh oh ho Sing Asia oh oh oh We will survive, yes. We will survive, together you and I We will survive coronavirus. We will survive. Together we will overcome. It doesn't matter anywhere you from. We will survive, Coronavirus. We will survive, together you and I. We will survive. We will survive, yes **(#23) English**	
Soldiers in green but corona looks like it's in patrol. But wait wait God will never fail. His holy name I hail. He go give we bail, bail to conquer. But wait wait, he is the king of kings. He never lose he always wins … We go conquer, we go conquer. Ebola came, we conquered it. Corona dey, we no dey shake. We're staying safe, build up our faith. Pray everyday cos we go conquer **(#2) English and GhPE**	“Soldiers in green but coronavirus looks like it's in patrol. But wait wait God will never fail. His holy name I hail. He will give us bail, bail to conquer. But wait wait, he is the king of kings. He never lose he always wins … We will conquer, we will conquer. Ebola came, we conquered it. Coronavirus exists, we are not afraid. We're staying safe. Build up our faith. Pray everyday because we will conquer”

## Discussion

As evident in Table 1, the lyrics analyzed in this study involved various topics to create awareness about COVID-19. The major themes found from the analysis are “public health guidelines;” “COVID-19 is real and not a hoax;” “COVID-19 is infectious;” “prayer as method to stop the virus;” “emotional responses and disruption of “everyday” activities;” “verbally expelling the virus;” “call for unity and collective efforts;” and “inspiring hope.” The lyrics provided content on the prevalence of the disease, how indiscriminating and infectious it is, and how it has disrupted social activities on both personal and global level. Some signs and symptoms were identified in the lyrics, along with steps that people can take to prevent the transmission of the disease.

The song lyrics tend to identify COVID-19 as a global social crisis with significant public health impact rather than a local health issue. Thus, some of the musicians resorted to prayer and asked for divine intervention. They inform the public that COVID-19 has no cure, and infected people may be asymptomatic. They also conveyed emotional appeals that warned listeners that the disease is “an (common) enemy,” “highly infectious,” “dangerous,” “wicked,” “scary,” “serious,” and “deadly.” While some of these descriptions may induce fear, the message that many people all over the world have been infected irrespective of age, race, nationality, physical stature or social status, can be viewed as an essential step in de-stigmatizing the disease.

Acknowledging that loneliness is a common feeling during social isolation can help to address mental health issues, and the act of inspiring hope in some lyrics may also enhance the quality of life of persons who are already infected (54) or assure listeners who are not yet infected that overcoming the disease is possible. The theme of inspiring hope in this study highlights a belief among Ghanaians that reassurance is needed in every predicament, and thus important in health and social care (40). Together, these songs propose a collective effort to completely fight the virus, but at the same time encourage listeners to take responsibility for their own health and adopt the necessary precautions to avoid the impact of the disease. The theme of “call for unity and collective efforts” reflects the communal nature of the Ghanaian society. The collectivity that is projected in the lyrics of the songs implicate not only those who are affected (in)directly by the disease but also, everyone around them (41, 42).

In addition to promoting the public health guidelines for COVID-19, some of the musicians encouraged the use of prayer as well as verbally expelling the virus as methods of combating the disease. This is unsurprising because research has revealed that in Ghana, some people conceptualize the causes and cures of diseases through both biomedical and religious/spiritual means (43, 44, 56). As noted by Okyerefo and Fiavi (56), among Ghanaians, the religious/spiritual methods are viewed as complementary rather than challenging or competing with the biomedical models of healthcare. Seeking divine intervention through prayer during the COVID-19 pandemic can be attributed to the belief that “doctors can treat certain conditions, but only God heals” ((56), p. 308). The act of verbally expelling the virus is a display of belief in the power of words among Ghanaians. The general notion is that the power of spoken words just as the health of a person has a link with the metaphysical and supernatural world. As a result, words can be effective in dismissing diseases and healing practices (45). In a broad sense, the themes of prayer and verbally expelling the virus in this study provide us with the understanding that in Ghana, biomedical models exist and operate alongside other healthcare practices.

The multilingual nature of Ghana was also reflected in the song lyrics. Akan, English, and GhPE were the languages used most often in the composition of these songs. This finding is not surprising, rather it validates the assertion that these languages serve as “*lingua francas*” in Ghana. Akan is the most widely used local language in different social contexts (46) while English is the defacto official/national language and a cross-ethnic “*lingua franca*” in Ghana (35, 36). GhPE, however, functions as the only medium of communication between English speakers and non-English speakers who do not share a common local language or non-English people of different linguistic backgrounds (47, 51). Most of the musical artists displayed their bilingual/multilingual identity as Ghanaian and primarily used these languages of wider communication to ground their messages in socio-culturally relevant contexts.

Even though there are several local languages spoken in sub-Saharan Africa (48), most of the WHO and national educational guidelines on COVID-19 are in the country's official language (English or French). It is therefore important for public health specialists, politicians, media experts, and social leaders to employ strategies that use local languages rather than an official language only understood by smaller portions of a country's population. The use of songs composed in local languages could be an important means of sensitizing vulnerable citizens to the awareness and health implications of COVID-19. Music has much to offer to the communication efforts related to COVID-19 and is especially appropriate for educational needs due to its inherent participatory nature (see (49)). In addition to musicians, other artistic entertainers such as comedians and poets should also be involved in the broader efforts to engage and educate the public through messages conveyed in their spoken words. This should be done in consultation with public health workers to avoid misinformation.

Despite educational efforts targeted at creating awareness about COVID-19, the number of cases continues to soar in many parts of the world. This increase would suggest the need for new and novel approaches that can complement the existing mechanisms for raising COVID-19 awareness and prevention. Song lyrics may be one such approach. Although some lyrics may be controversial or irrelevant just for the purpose of creating a rhythm, music still represents an inexpensive yet innovative and powerful tool for discussing the novel coronavirus and best practices for preventing its spread. It is also noteworthy that not all song lyrics will make a positive contribution to promoting preventive behaviors; however, analysis of song lyrics can provide insight into local attitudes toward the virus. In addition, collaboration between health workers and musicians can offer a means to disseminate information in an accessible way.

Undoubtedly, additional research is needed in order to fully understand the impact of using music and lyrics to educate the public on COVID-19, however existing literature clearly demonstrates that music contributes a great deal to public education efforts related to HIV/AIDS and other infectious diseases. The messages embedded in the lyrics of songs are designed to reach a large segment of the population faster and in a meaningful and more memorable fashion. People with a broad range of literacy and access to information can better retain these types of messages and thus achieve the goal of public health knowledge.

## Limitations

The major limitations of this study include the limited focus on Ghana music and the potential for missing some songs created during this short time frame or not having access to other songs that were short-lived in the media. In addition, many of the songs used as data for this study could benefit from a more detailed lyrical analysis (9), which we hope will be undertaken in future research. Nevertheless, this paper focused on the unique and novel contribution of music to creating awareness about COVID-19 in Ghana and similar studies should also be conducted in other countries. Also, we did not assess the impact of the song lyrics on attitudes, beliefs, and behaviors; therefore, a more comprehensive ethnographic study is needed to understand the impact of the songs on the society.

## Conclusion

Raising public awareness on any social issue or health crisis often demands an increase in information dissemination through both formal and informal means. While it was not our intention to prioritize any one dissemination strategy, educating the public through diverse musical genres and other non-deliberative means could yield valuable global results. Music is one such information dissemination vehicle or promotion strategy utilized in health communication in many places over time, mainly because it has the potential to facilitate the process of behavioral change among specific sectors of the population. We showed that the lyrics in the songs used as data are informative and encourage people that they can and should take responsibility for their health and adhere to necessary safety protocols. Thus, the discourse on COVID-19 expressed through songs has relevance for the development of culturally appropriate health messaging. Results from this study support the idea that music has the ability to contribute to the fight against coronavirus and can be employed as an enjoyable supplement that reinforces or amplifies educational efforts in various communities around the world.

Results from this study can also be used to inform policymakers that investing in health communications using music could be worthwhile. Supporting musicians with sufficient resources to engage and educate citizens about the ongoing pandemic could be cost-effective. This study should be viewed as the beginning of a journey to document how music can be used to raise awareness about COVID-19. Generally, music is not immune to the problems of misinformation, and attention to song lyrics may provide broad insight into local discourse, including ideas that may contradict accepted public health messages. Therefore, a future study could focus on some recognition of the diversity of messages that may be communicated through songs in order to provide a more nuanced perspective. Further research is required to assess the impact of song lyrics as well as melodies and rhythms. However, results from this study should stimulate interest in how to use songs and tailor them to local communities to fight against COVID-19 and other future pandemic health crises.

## Data Availability Statement

The original contributions presented in the study are included in the article/Supplementary Materials, further inquiries can be directed to the corresponding author/s.

## Author Contributions

RT participated in the design of the study, analyzed all the research data, and drafted the manuscript. JN participated in the design of the study, analyzed the data, and reviewed the manuscript. JJ reviewed the manuscript and supervise the study. All authors read and approved the final manuscript.

## Conflict of Interest

The authors declare that the research was conducted in the absence of any commercial or financial relationships that could be construed as a potential conflict of interest.
