# Supplementary material for: Communicating Awareness About COVID-19 Through Songs: An Example From Ghana
Source: Front Public Health. 2021 Jan 18;8:607830. doi: 10.3389/fpubh.2020.607830 (PMC7847897; doi:10.3389/fpubh.2020.607830)
Supplement: Supplementary file 1 [file Table_1.docx]

***Supplementary Table***

**Table 1**: List of 28 Compiled Songs

| Song Title | Artist(s) |
| --- | --- |
| 1. Beware of Corona Virus | Obiba Sly featuring Mawunya |
| 1. Conquer | King Eazie \| Besa \| KLord |
| 1. Coromental | Reggie ‘N’ Bollie |
| 1. Corona | Abochi featuring Dede Supa |
| 1. Corona | Kweku Smoke |
| 1. Corona | Tulenkey |
| 1. Corona Jam | Wiyaala and family |
| 1. Corona | Sister Afia |
| 1. Corona Virus | Patapaa |
| 1. Corona Virus | Cryme Officer |
| 1. Corona Virus | Kojo-K |
| 1. Coronavirus | Ahkan |
| 1. Corona virus | Ama Grace |
| 1. Corona virus | Article Wan |
| 1. Corona virus | Eno Barony |
| 1. Coronavirus | Fancy Gadam |
| 1. COVID-19 | Bless |
| 1. COVID-19 | Nana Ampadu featuring the Children of J. A. Obuoba, Adofo, & Nana Ampadu |
| 1. COVID-19 | Justice_Man aka Bunfyah Dube |
| 1. Lockdown | Zanli |
| 1. Lockdown COVID-19 | Slim Buster |
| 1. Stay Home, Stay Safe | Naa Ashorkor X Clemento Suarez featuring Deelaw |
| 1. We will Survive (Corona Anthem) | Kobi Rana |
| 1. Aunty Coro | Rolly Panda |
| 1. Corona Virus | Badwenba |
| 1. Corona Virus Awareness in Ghana | Obeng Acheampong and Bomaa Paradise Singers |
| 1. Corona Virus (Coro’Coro) | Kamelyeon |
| 1. Coronavirus | Myka featuring Evance |
